# Prevalence and factors associated with high arsenic exposure in the adult population of Chumbivilcas in Cusco, Peru 2024

**DOI:** 10.17843/rpmesp.2026.433.16608

**Published:** 2026-09-29

**Authors:** Alex Jaramillo-Corrales, Ruth Nelly Oscco-Abarca, Eduarda Benito-Calderón, Milton Cesar Pechuga-Melgar, Guinetta Margarita Yabar-Herrera, Maribel Caller-Soto, Deisy Clarivel Paredes-Herrera, Beatriz Huayhua-Sivincha, Joan Miguel Ángel Castro-Benito, José Santa Cruz-Quispe

**Affiliations:** 1 Dirección de Epidemiología e Investigación, Dirección Ejecutiva de Inteligencia Sanitaria, Gerencia Regional de Salud Cusco, Ministerio de Salud, Cusco, Peru. Dirección de Epidemiología e Investigación, Dirección Ejecutiva de Inteligencia Sanitaria Gerencia Regional de Salud Cusco Ministerio de Salud Cusco Peru; 2 Escuela Profesional de Medicina Humana, Facultad de Medicina Humana, Universidad Nacional de San Antonio Abad del Cusco, Cusco, Peru. Universidad Nacional San Antonio Abad del Cusco Escuela Profesional de Medicina Humana Facultad de Medicina Humana Universidad Nacional de San Antonio Abad del Cusco Cusco Peru; 3 Unidad de Epidemiología, Dirección de Inteligencia Sanitaria, Red de Servicios de Salud Chumbivilcas, Cusco, Peru. Unidad de Epidemiología Dirección de Inteligencia Sanitaria Red de Servicios de Salud Chumbivilcas Cusco Peru; 4 Dirección de Salud de las Personas, Dirección Ejecutiva de Atención Integral de Salud, Gerencia Regional de Salud Cusco, Ministerio de Salud, Cusco, Peru. Dirección de Salud de las Personas, Dirección Ejecutiva de Atención Integral de Salud Gerencia Regional de Salud Cusco Ministerio de Salud Cusco Peru; 5 Centro de Salud de Livitaca, Red de Servicios de Salud Chumbivilcas, Cusco, Peru. Centro de Salud de Livitaca Red de Servicios de Salud Chumbivilcas Cusco Peru; 6 Dirección de Laboratorio de Referencia Regional de Salud Pública, Gerencia Regional de Salud Cusco, Ministerio de Salud, Cusco, Peru. Dirección de Laboratorio de Referencia Regional de Salud Pública Gerencia Regional de Salud Cusco Ministerio de Salud Cusco Peru

**Keywords:** Arsenic, Environmental exposure, Drinking Water, Water pollution, Heavy metals and Occupational exposure

## Abstract

**Objectives.:**

To determine the prevalence and factors associated with high arsenic exposure in the adult population of the province of Chumbivilcas in Cusco, Peru 2024.

**Materials and methods.:**

Analytical cross-sectional study in 370 adults selected through stratified cluster probability sampling by district. The urinary arsenic concentration was determined by inductively coupled plasma mass spectrometry (ICP-MS) and adjusted for creatinine; high exposure was defined as a concentration ≥20 µg As/g of creatinine. Sociodemographic and occupational characteristics, epidemiological history of exposure, and environmental factors were evaluated as potential associated factors. Adjusted prevalence ratios (aPR) with 95% confidence intervals (95% CI) were estimated using Poisson regression models with a log link.

**Results.:**

The prevalence of high arsenic exposure was 68.2% (95% CI: 61.9-73.9). Exposure at work was associated with a higher prevalence of high arsenic (aPR 1.29; 95% CI: 1.07-1.56), as was the consumption of spring water (aPR 1.19; 95% CI: 1.01-1.40). Exposure at home (aPR 0.83; 95% CI: 0.69-0.99) and the respiratory route (aPR=0.82; 95% CI: 0.72-0.92) showed a lower prevalence of high arsenic.

**Conclusions.:**

The adult population of Chumbivilcas presented a high prevalence of arsenic exposure. Exposure at work and the use of spring water were associated with a higher prevalence. Studies incorporating environmental measurements are required to guide surveillance and prevention actions.

## INTRODUCTION

Arsenic is a metalloid widely distributed in the Earth's crust, whose environmental presence can originate from both natural processes and anthropogenic activities. In volcanic and geothermal regions, particularly relevant in the Andes, the weathering and leaching of minerals and rocks can favor its mobilization into surface and groundwater; likewise, activities such as mining and smelting can contribute to its release into the environment ^1^^,^^2^. In this context, environmental exposure to arsenic constitutes a global public health problem. In the population, the main route of exposure is the ingestion of contaminated water and food ^3^.

The World Health Organization considers arsenic a chemical contaminant of public health importance due to its toxicity and the exposure of populations through drinking water ^3^. Chronic exposure to arsenic has been associated with adverse health effects, including skin, lung, bladder, and kidney cancer, as well as cardiovascular and metabolic diseases ^4^^-^^6^. Available evidence indicates an increased risk of mortality associated with prolonged exposure to this metalloid ^4^^,^^6^. In Latin America, the presence of arsenic in water sources has been widely documented, especially in regions with mining activity or with volcanic and sedimentary geological characteristics. Regional reviews have described the existence of areas with elevated concentrations of arsenic in groundwater, affecting populations in countries such as Argentina, Chile, Bolivia, Mexico, and Peru. These conditions have been primarily associated with natural processes of geochemical mobilization, as well as anthropogenic activities, particularly mining ^7^^-^^9^.

In Peru, exposure to heavy metals and metalloids has been recognized as an emerging public health problem. Various studies have documented the presence of arsenic in water sources intended for human consumption, particularly in rural and high-Andean areas. George *et al*. showed that a significant proportion of water sources in Peruvian communities exceed the provisional guideline value of 10 µg/L recommended by WHO for arsenic in drinking water, which represents a risk to the health of exposed populations ^10^. Likewise, studies in mining areas have shown the presence of metals in human biomarkers, reflecting sustained environmental exposure ^11^.

In the Andean context, arsenic exposure acquires particular relevance due to the combination of environmental, geological, and socioeconomic factors that condition access to safe water. In these populations, the dependence on untreated water sources, together with proximity to mining activities, can increase the risk of chronic exposure. Studies conducted in Andean populations have reported elevated arsenic concentrations in water sources and in urine, as a biomarker of recent exposure ^12^.

As a local precedent, in 2023 the Chumbivilcas Health Services Network conducted a targeted evaluation of metal exposure in 80 residents from two localities previously identified as having potential risk: the Llanque quarry area, in the district of Colquemarca, and the district of Quiñota. By determining arsenic in urine, 78 of the 80 evaluated individuals (97.5%) presented concentrations ≥20 µg As/g of creatinine, taking as a reference the value established for non-occupationally exposed persons in the Technical Guide for the Comprehensive Approach to Persons Exposed to Arsenic of the Ministry of Health ^(^^13^. The selection of the evaluated individuals was not probabilistic and was focused on localities previously identified as being at potential risk; therefore, these results constituted a health signal of exposure and not an estimate of population prevalence. Consequently, the need persisted to estimate the magnitude of exposure through a probabilistic study with provincial coverage in the population of Chumbivilcas. In this context, the present study aimed to determine the prevalence and factors associated with elevated arsenic exposure in inhabitants of the province of Chumbivilcas, Peru, during 2024.

**KEY MESSAGES**

Motivation for the study. In Chumbivilcas, there is limited population evidence based on biomarkers regarding the magnitude of arsenic exposure and its associated factors.

Main findings. A high prevalence of arsenic exposure was found in the adult population of Chumbivilcas. Reported occupational exposure and spring water use were associated with a higher prevalence.

Public health implications. The findings support the need to strengthen epidemiological and environmental surveillance in Chumbivilcas and to deepen the evaluation of potential sources of exposure, especially in the workplace and water sources, through studies that incorporate environmental measurements.

## MATERIALS AND METHODS

### Design and study population

An observational, cross-sectional, analytical study was conducted. The study was carried out in the province of Chumbivilcas, located in the Cusco region, in southern Peru, at an average altitude of 3,660 meters above sea level. This province has a predominantly rural geography, and its main economic activities include agriculture, livestock farming, mining, and local trade. The projected population for 2024 was 69,017 inhabitants across its 8 districts ^14^. Participants over 18 years of age, of both sexes, who had resided in the area for a minimum period of six months prior to data collection, were included; and those who had been absent for more than 10 consecutive days in the last month, had any cognitive disability, had consumed fish or shellfish 3 days before, and individuals with chronic kidney disease or who held any political, public, or representative position were excluded.

### Sampling and sample size

A two-stage cluster probability sampling was used, stratified by districts of the province. In the first stage, population clusters within each district were selected, and in the second stage, households within each cluster were selected. Subsequently, eligible adult residents of the selected households were invited to participate. The final sample size consisted of 370 participants in 25 clusters, with districts having one cluster such as Quiñota and others having nine clusters such as Santo Tomás, who met the eligibility criteria and agreed to participate in the study.

### Study variables

The dependent variable was arsenic exposure, determined by urinary arsenic concentration adjusted for creatinine. Exposure was considered when the concentration was equal to or greater than 20 µg/g of creatinine, according to criteria established in the international literature and adopted by the National Center for Occupational Health and Environmental Protection for Health (CENSOPAS) of the National Institute of Health (INS) ^15^.

The independent variables that, act as sociodemographic, epidemiological, and environmental factors were recorded using the Public Health Epidemiological Research Form for Risk Factors due to Exposure and Intoxication by Heavy Metals and Metalloids from the Ministry of Health ^16^. The form records the route of exposure as respiratory, oral, dermal, unknown, or other, and the type of exposure as occupational, accidental, environmental, or other. These variables corresponded to epidemiological history recorded using the form and not to direct measurements of arsenic in environmental matrices. On the other hand, the proximity of the dwelling to mining, mining tailings, and transport of metal concentrates corresponded to the dichotomous record (yes/no) established in the form, without specific distance measurement. Likewise, the presence of dust in the external environment was recorded dichotomously (yes/no), without instrumental measurement of particulate matter. The use of any non-potable water source was defined as the reported consumption of river, spring, or rainwater, independently of the concomitant use of potable sources.

### Procedures

The fieldwork was conducted for seven consecutive days, from July 22 to 28, 2024, including weekdays and weekends. Home visits were conducted between 6:30 and 14:00 hours. In each household, the participant was randomly selected from among eligible adults. When the selected person was not at home or refused to participate, another eligible adult from the same household was selected, and, when this was not possible, an adult from the adjacent household. The interview and urine sample collection were conducted during the same home visit.

The urine samples were kept under refrigeration at the Santo Tomás Hospital, Chumbivilcas, following the conditions established in the CENSOPAS-INS procedure, which establishes a preservation temperature between 2 and 8 °C for the determination of arsenic in spot urine. For their transfer from Chumbivilcas to the city of Cusco, *coolers* with refrigerant gels were used, keeping the samples refrigerated during transport. Subsequently, they were transported by air from Cusco to Lima for reception at CENSOPAS-INS. The shipment was carried out in two batches, on July 25 and 29, 2024, during the fieldwork period, in order to keep the samples within the established conditions and times for their preservation.

Samples were processed at the Chemical Toxicology Laboratory of CENSOPAS in Lima, Peru. Urinary arsenic concentration was determined by inductively coupled plasma mass spectrometry (ICP-MS), using method MET-CENSOPAS-017 “Determination of arsenic and cadmium in human urine samples by inductively coupled plasma mass spectrometry”. Results were reported in µg/L and normalized by urinary creatinine in µg As/g creatinine.

### Statistical analysis

For the analysis, the complex sample design of the study was considered, incorporating the sample weights, stratification by district, and clustering. The prevalence of high arsenic exposure was estimated considering the sample design and presented with its 95% confidence interval (95% CI). To evaluate associated factors, crude and adjusted prevalence ratios (aPR) were estimated, with their respective 95% CI, using Poisson regression models with a log link that incorporated the sample design. The aPR for age, sex, and origin were obtained using a mutually adjusted model. For occupational, epidemiological, and environmental variables, independent models adjusted for age, sex, and origin were used, with the purpose of maintaining parsimonious models based on the available degrees of freedom of the sample design. A p-value <0.05 was considered statistically significant. Statistical analysis was performed using R version 4.5.2 (R Foundation for Statistical Computing, Vienna, Austria).

### Ethical aspects

The study was approved by the Research Ethics Committee of the Universidad Nacional de San Antonio Abad del Cusco, through Report No. 007-2024.CBI-UNSAAC. All participants received information about the study objectives and granted their informed consent prior to their participation. Likewise, the confidentiality of the collected information was guaranteed and the use of the data exclusively for research purposes.

## RESULTS

A total of 370 participants residing in the eight districts of the province of Chumbivilcas were included. Of these, 250 had urinary arsenic concentrations adjusted for creatinine ≥20 µg As/g creatinine and 120 had values below this cutoff point. Considering the sampling design, the weighted prevalence of high arsenic exposure was 68.2% (95% CI: 61.9-73.9). The point estimates of weighted prevalence by district ranged between 56.9% in Santo Tomás and 86.7% in Capacmarca and Chamaca.

The characteristics of the studied population are presented in table 1. The mean age was 46.2 years (SE: 1.2); 59.4% were female, and 64.5% came from rural areas. Regarding educational level, secondary (34.6%) and primary (32.1%) education predominated. The most frequent occupations were agricultural activities (36.1%) and housework (34.2%).

**Table 1:** Characteristics of arsenic exposure in adults from Chumbivilcas province, Cusco 2024.

Characteristics		n	(%)^a^
Age, years^b^		370	46.2 (1.2)
Sex			
	Female	217	(59.4)
	Male	153	(40.6)
Origin			
	Urban	125	(32.6)
	Urban-marginal	12	(2.9)
	Rural	233	(64.5)
District^c^			
	Capacmarca	15	(86.7)
	Chamaca	30	(86.7)
	Colquemarca	45	(68.9)
	Livitaca	60	(76.7)
	Llusco	30	(60.0)
	Quiñota	15	66.7
	Santo Tomás	130	(56.9)
	Velille	45	(71.1)
Educational level			
	Preschool	4	(1.1)
	Primary	117	(32.1)
	Secondary	131	(34.6)
	Superior	65	(17.2)
	Without instruction	53	(15.0)
Occupation			
	Public servant	29	(7.9)
	Self-employed	48	(13.0)
	Student	2	(0.4)
	Mining	32	(8.4)
	Homemaker	125	(34.2)
	Agricultural	134	(36.1)
Health insurance			
	SIS	353	(95.5)
	EsSalud	17	(4.5)
Water source^d^			
	Network	303	(83.0)
	Public tap	25	(6.3)
	River	33	(8.6)
	Spring	48	(12.1)
	Rainwater	7	(1.7)
	Any non-potable source^e^	84	(21.5)
Urinary arsenic concentration^f^			
	Mean (SE)	327	39.39 (2.24)
	Median (IQR)	327	29.02 (20.44-44.75)
Elevated exposure to arsenic^g^			
	No	120	(31.8)
	Yes	250	(68.2)

a The estimates incorporated weighting, stratification, and clustering of the sample design; ^b^ Mean (standard error); ^c^ Point estimates of arsenic in the districts. Confidence intervals are not presented due to the reduced number of clusters, which prevented their estimation with reliable precision due to their comparability; ^d^ Water sources were not mutually exclusive; ^e^ Water reported from river water, spring, or rain was considered a non-potable source; ^f^ Arsenic concentration was quantifiable in 327 participants and expressed in µg As/g creatinine; urinary arsenic concentration was quantified as mean (standard error) and as median (interquartile range); ^g^ High exposure was considered a urinary concentration ≥20 µg As/g creatinine; results were not stratified as unexposed.

The results of the adjusted analyses found no factors associated with high arsenic exposure regarding the associated sociodemographic and occupational characteristics (table 2).

**Table 2:** Sociodemographic and occupational factors associated with high arsenic exposure in adult residents of Chumbivilcas Province, Cusco 2024.

Characteristics		Crude PR (95%CI)	“ Model 1 aPR (95% CI) ”	“ Model 2 aPR (95% CI) ”	“ Model 3 aPR (95% CI) ”
Age, years		0.99 (0.98-1.02)	0.995 (0.98-1.00)		
Sex					
	Female	Ref.	Ref.		
	Male	0.88 (0.77-1.02)	0.90 (0.78-1.05)		
Origin					
	Urban	Ref.	Ref.		
	Urban-marginal	1.13 (0.89-1.44)	1.12 (0.85-1.46)		
	Rural	1.23 (0.96-1.59)	1.26 (0.98-1.62)		
Occupation					
	Public servant	Ref.	—	Ref.	—
	Sef-employed	1.35 (0.90-2.04)	—	1.36 (0.92-2.00)	—
	Student	1.02 (0.15-6.70)	—	0.90 (0.17-4.84)	—
	Mining	1.48 (0.92-2.38)	—	1.58 (0.96-2.59)	—
	Homemaker	1.43 (0.90-2.27)	—	1.30 (0.84-2.01)	—
	Agricultural	1.43 (0.89-2.31)	—	1.51 (0.95-2.39)	—
Economic activity					
	Formal mining	Ref.	—	—	Ref.
	Informal mining	0.78 (0.66-0.94)	—	—	0.81 (0.66-0.98)
	Transport of metals	0.87 (0.48-1.59)	—	—	0.92 (0.46-1.83)
	Welding	0.60 (0.14-2.61)	—	—	0.76 (0.15-3.68)
	Agricultural	0.74 (0.61-0.90)	—	—	0.78 (0.64-0.96)

Model 1 corresponds to the mutual adjustment between age, sex, and origin. Model 2 evaluates occupation adjusting for age, sex, and origin. Model 3 evaluates economic activity adjusting for age, sex, and origin. According to the foot of the original table, all models incorporated weighting, stratification, and clustering of the sample design. Ref.: reference category.

Table 3 shows that the exposure reported at work was associated with a higher prevalence of high arsenic exposure (aPR=1.29; 95% CI: 1.07-1.56). In contrast, the respiratory route of exposure presented a lower prevalence (aPR=0.82; 95% CI: 0.72-0.92). No associations were observed with the other evaluated epidemiological variables. Table 4 shows that the consumption of spring water was associated with a higher prevalence of high arsenic exposure (aPR=1.19; 95% CI: 1.01-1.40). No associations were observed with the use of any non-potable water source or with the other evaluated environmental variables.

**Table 3:** Association between epidemiological indicators and high arsenic exposure in adult residents of Chumbivilcas Province, Cusco 2024.

Characteristics		PR (95% CI)	aPR (95% CI)*
Reported exposure at home			
	No	Ref.	Ref.
	Yes	0.86 (0.73-1.01)	0.83 (0.69-0.99)
Exposure reported at work			
	No	Ref.	Ref.
	Yes	1.21 (1.04-1.39)	1.29 (1.07-1.56)
Reported airway			
	No	Ref.	Ref.
	Yes	0.77 (0.66-0.90)	0.82 (0.72-0.92)
Reported oral route			
	No	Ref.	Ref.
	Yes	1.08 (0.93-1.25)	1.06 (0.84-1.34)
Unknown pathway			
	No	Ref.	Ref.
	Yes	0.92 (0.74-1.15)	0.98 (0.77-1.25)
Smoking habit			
	No	Ref.	Ref.
	Yes	1.17 (0.89-1.54)	1.14 (0.86-1.52)

Adjusted for age, sex, and origin. All models incorporated weighting, stratification, and clustering of the sample design. Home and work exposure corresponds to exposure reported by participants. Respiratory, oral, and unknown routes correspond to exposure routes reported by participants. Ref.: reference category.

**Table 4:** Association between environmental factors and elevated Arsenic exposure in adult residents of Chumbivilcas Province, Cusco 2024.

Characteristic		PR (95% CI)	aPR (95% CI)^a^
Proximity to mining activity			
	No	Ref.	Ref.
	Yes	1.07 (0.90-1.26)	1.04 (0.90-1.21)
Proximity to mining tailings			
	No	Ref.	Ref.
	Yes	1.06 (0.80-1.41)	1.01 (0.74-1.37)
Proximity to metals/minerals transport			
	No	Ref.	Ref.
	Yes	1.22 (0.75-1.98)	1.20 (0.72-2.02)
Proximity to the corridor			
	No	Ref.	Ref.
	Yes	0.86 (0.63-1.19)	0.82 (0.60-1.13)
Presence of dust outdoors			
	No	Ref.	Ref.
	Yes	0.93 (0.54-1.59)	0.97 (0.58-1.63)
Use of any non-potable water source			
	No	Ref.	Ref.
	Yes	1.04 (0.81-1.34)	0.99 (0.78-1.24)
Use of Spring Water			
	No	Ref.	Ref.
	Yes	1.25 (1.05-1.49)	1.19 (1.01-1.40)

a Adjusted for age, sex, and origin. All models incorporated weighting, stratification, and clustering of the sample design. Non-potable water source was defined as the reported use of at least one of the following sources: river, spring, or rainwater; water sources were not mutually exclusive. Spring water use was additionally evaluated as a specific water source. Ref.: reference category.

## DISCUSSION

In this population-based study conducted in the province of Chumbivilcas, a high weighted prevalence of arsenic exposure was found (68.2%; 95% CI: 61.9-73.9). Reported occupational exposure and spring water use were associated with a higher prevalence of high arsenic exposure. In contrast, the recorded respiratory route showed an inverse association. No association was found with the use of any non-potable water source or with occupation.

The magnitude of exposure observed in Chumbivilcas is consistent with the heterogeneity described in Latin America, where arsenic contamination affects both groundwater and surface water and presents significant geographical variations, particularly in Andean regions with geological conditions and mining activities that can favor its mobilization ^1^^,^^2^.

In Peru, Ale-Mauricio *et al*. reported in Cairani and Camilaca, Tacna, median urinary arsenic levels of 601.6 and 30.2 µg/g of creatinine, respectively, with 100% and 80.3% of participants above 20 µg/g of creatinine; in these districts, the water supply was described as being of underground origin, with contributions from the Callazas River and the Japo spring ^17^. Likewise, Fano-Sizgorich *et al*. found a mean total urinary arsenic level of 43.97 µg/L in pregnant women from Tacna and indicated that, in a previous evaluation of this population, 70.2% were exposed to arsenic concentrations in drinking water ≥25 µg/L ^18^.

In Bolivia, De Loma *et al*. reported a median urinary arsenic of 65 µg/L in women from communities near Lake Poopó, where elevated arsenic concentrations were documented in drinking water sources ^12^. These differences between populations could be related to the geological heterogeneity of the Andean region, the sources and magnitude of exposure, and the characteristics of the studied populations ^1^^,^^2^^,^^12^. In Chumbivilcas, although the use of spring water was associated with a higher prevalence of elevated arsenic exposure, the use of any non-potable water source showed no association; therefore, our results do not allow attributing the observed high prevalence to drinking water in general.

The magnitude of exposure observed was lower than that reported in populations specifically selected for their environmental exposure, which should be interpreted considering the population-based approach of our study. High arsenic exposure was present both in participants who reported residing near mining activity and in those who did not, with no association evidenced with this condition.

Exposure reported at work was associated with a higher prevalence of high arsenic exposure (aPR=1.29; 95% CI: 1.07-1.56), which is compatible with evidence recognizing occupational environments and activities linked to mining and metals as possible exposure scenarios ^1^^,^^2^^,^^15^^,^^19^. In contrast, the reported household exposure showed an inverse association (aPR=0.83; 95%CI: 0.69-0.99). This result should be interpreted with caution, as both variables correspond to historical data recorded via the epidemiological record form and not to environmental arsenic measurements; therefore, they do not allow for the identification of the specific location or source of exposure.

The use of spring water was associated with a higher prevalence of high arsenic exposure (aPR=1.19; 95% CI: 1.01-1.40). This finding is consistent with the relevance of water sources as an exposure route described in Peru and other Andean contexts. George *et al.* documented arsenic concentrations greater than 10 µg/L in 86% of the groundwater samples evaluated in different localities in Peru ^10^. Likewise, García-Rico *et al.* documented arsenic in drinking water and soils of communities in Sonora, Mexico, and found a positive correlation between urinary arsenic levels and the environmental risk index ^20^; In the Chilean Altiplano, elevated arsenic concentrations have been described in surface waters, related to the geochemical characteristics of the region ^21^. The presence of arsenic in water sources in Latin America may be due to various geogenic and anthropogenic processes, with particular relevance of the Andean geological conditions ^1^. However, in our study, the use of any non-potable water source did not show an association with elevated arsenic exposure, so the specific finding for spring water does not allow establishing that water is the causal source of exposure, given that arsenic concentrations were not measured in the water sources used by the participants.

The respiratory route recorded in the epidemiological record was associated with a lower prevalence of high arsenic exposure (aPR=0.82; 95% CI: 0.72-0.92). This finding must be interpreted with caution and does not imply a protective effect of respiratory exposure. The variable corresponded to the history of exposure route documented through the epidemiological record and not to an air arsenic measurement nor to a quantitative assessment of inhalation exposure. Although inhalation can constitute a relevant route of arsenic exposure in certain occupational and environmental contexts ^1^^,^^2^^,^^22^, our study does not allow establishing a mechanism that explains the observed inverse association. Therefore, this result could reflect differences in the exposure profiles or sources of the participants and requires evaluation in studies that incorporate specific environmental measurements.

The differences observed between our results and those reported in other studies can be explained by variations in hydrogeological conditions, the types of biomarkers used, the cut-off points employed, and the characteristics of the populations evaluated. Furthermore, studies conducted in specific areas with high contamination tend to report higher levels of exposure compared to population-based studies.

From a public health perspective, our findings are relevant due to the multiple health consequences associated with chronic arsenic exposure. Epidemiological evidence has shown that prolonged exposure is associated with an increased risk of cancer, cardiovascular diseases, and neurological disorders ^(4-6)^. In this context, a high prevalence like that observed in our population suggests a potential risk of cumulative chronic disease burden.

This study presents some limitations. Firstly, arsenic measurements were not performed in water, air, soil, food, or other environmental matrices, so the observed associations do not allow identification of the specific source of exposure. Epidemiological and environmental variables were obtained using the investigation form and did not represent quantitative measurements of exposure intensity or dose, so they are susceptible to classification errors and information bias. Finally, the limited number of clusters reduced the degrees of freedom available for analysis and restricted the complexity of multivariate models; consequently, independent parsimonious models adjusted for age, sex, and origin were used, without residual confounding being ruled out. This limitation also affected the precision of estimates by district.

Although the fieldwork included weekdays and weekends and the visits started from 6:30 a.m., which expanded the opportunity to include occupationally active people before the start of their usual activities, the replacement of selected participants who were absent or refused to participate by other adults from the same household or, when this was not possible, from an adjacent household, could have modified the individual probabilities of selection and generated selection bias. It is not possible to establish the direction or magnitude of this potential bias on the estimates obtained.

Among the strengths of the study, its population-based approach stands out, with the inclusion of participants from the eight districts of the province of Chumbivilcas through stratified cluster probability sampling by district. The analysis incorporated the weighting, stratification, and clustering of the sampling design, allowing population estimates to be obtained and the sample structure to be considered in estimating its precision. Likewise, high exposure was determined by measuring urinary arsenic, providing an objective measure of the exposure biomarker. Finally, the joint evaluation of sociodemographic, epidemiological, and environmental characteristics allowed exploring different factors in the general population and generating evidence to guide subsequent environmental and epidemiological research.

In conclusion, in the studied population of Chumbivilcas, a high weighted prevalence of arsenic exposure was found. Origin showed an overall association with high exposure, while reported occupational exposure and spring water use were associated with a higher prevalence; in contrast, reported household exposure and the respiratory route showed inverse associations that should be interpreted with caution. These findings do not allow for the establishment of causal sources or routes of exposure and highlight the need to strengthen epidemiological and environmental surveillance, as well as to develop studies that integrate biomonitoring and arsenic measurements in water and other environmental matrices to identify sources of exposure and guide prevention measures in Chumbivilcas.
