# Supplementary material for: Prevalence and factors associated with high arsenic exposure in the adult population of Chumbivilcas in Cusco, Peru 2024
Source: Rev Peru Med Exp Salud Publica. 2026 Sep 29;43(3):321–9. doi: 10.17843/rpmesp.2026.433.16608 (PMC13718572; doi:10.17843/rpmesp.2026.433.16608)
Supplement: Translation

# Translation: Prevalencia y factores asociados a la exposición elevada a arsénico en población adulta de Chumbivilcas en Cusco, Perú 2024

**Authors:** Alex Jaramillo-Corrales, Ruth Nelly Oscco-Abarca, Eduarda Benito-Calderón, Milton Cesar Pechuga-Melgar, Guinetta Margarita Yabar-Herrera, Maribel Caller-Soto, Deisy Clarivel Paredes-Herrera, Beatriz Huayhua-Sivincha, Joan Miguel Ángel Castro-Benito, José Santa Cruz-Quispe

**Affiliations:** 1 Dirección de Epidemiología e Investigación, Dirección Ejecutiva de Inteligencia Sanitaria, Gerencia Regional de Salud Cusco, Ministerio de Salud, Cusco, Perú.; 2 Escuela Profesional de Medicina Humana, Facultad de Medicina Humana, Universidad Nacional de San Antonio Abad del Cusco, Cusco, Perú.; 3 Unidad de Epidemiología, Dirección de Inteligencia Sanitaria, Red de Servicios de Salud Chumbivilcas, Cusco, Perú.; 4 Dirección de Salud de las Personas, Dirección Ejecutiva de Atención Integral de Salud, Gerencia Regional de Salud Cusco, Ministerio de Salud, Cusco, Perú.; 5 Centro de Salud de Livitaca, Red de Servicios de Salud Chumbivilcas, Cusco, Perú.; 6 Dirección de Laboratorio de Referencia Regional de Salud Pública, Gerencia Regional de Salud Cusco, Ministerio de Salud, Cusco, Perú.

**Keywords:** Arsénico, Exposición ambiental, Agua Potable, Contaminación del agua, Metales pesados y Exposición ocupacional

## Abstract

**Objetivos.:**

Determinar la prevalencia y los factores asociados a la exposición elevada a arsénico en población adulta de la provincia de Chumbivilcas en Cusco, Perú 2024.

**Materiales y métodos.:**

Estudio transversal y analítico en 370 adultos seleccionados mediante muestreo probabilístico por conglomerados estratificado por distrito. La concentración de arsénico urinario se determinó mediante espectrometría de masas con plasma acoplado inductivamente (ICP-MS) y se ajustó por creatinina; se definió exposición elevada como una concentración ≥20 µg As/g de creatinina. Como posibles factores asociados se evaluaron características sociodemográficas y ocupacionales, antecedentes epidemiológicos de exposición y factores ambientales. Se estimaron razones de prevalencia ajustadas (RPa) con intervalos de confianza al 95% (IC95%) mediante modelos de regresión de Poisson con enlace logarítmico.

**Resultados.:**

La prevalencia de exposición elevada a arsénico fue 68,2% (IC95%: 61,9-73,9). La exposición en el trabajo se asoció con mayor prevalencia de arsénico elevado (RPa 1,29; IC95%: 1,07-1,56), al igual que el consumo de agua de manantial (RPa 1,19; IC95%: 1,01-1,40). La exposición en el hogar (RPa 0,83; IC95%: 0,69-0,99) y la vía respiratoria (RPa=0,82; IC95%: 0,72-0,92) mostraron menor prevalencia de arsénico elevado.

**Conclusiones.:**

La población adulta de Chumbivilcas presentó una elevada prevalencia de exposición a arsénico. La exposición en el trabajo y el uso de agua de manantial se asociaron con una mayor prevalencia. Se requieren estudios que incorporen mediciones ambientales para orientar las acciones de vigilancia y prevención.

## INTRODUCCIÓN

El arsénico es un metaloide ampliamente distribuido en la corteza terrestre, cuya presencia ambiental puede originarse tanto por procesos naturales como por actividades antropogénicas. En regiones volcánicas y geotérmicas, particularmente relevantes en los Andes, la meteorización y lixiviación de minerales y rocas pueden favorecer su movilización hacia aguas superficiales y subterráneas; asimismo, actividades como la minería y la fundición pueden contribuir a su liberación al ambiente ^1^^,^^2^. En este contexto, la exposición ambiental al arsénico constituye un problema de salud pública a nivel mundial. En la población, la principal vía de exposición es la ingestión de agua y alimentos contaminados ^3^.

La Organización Mundial de la Salud considera al arsénico un contaminante químico de importancia para la salud pública debido a su toxicidad y a la exposición de poblaciones a través del agua de consumo ^3^. La exposición crónica a arsénico se ha asociado con efectos adversos sobre la salud, entre ellos cáncer de piel, pulmón, vejiga y riñón, así como enfermedades cardiovasculares y metabólicas ^4^^-^^6^. La evidencia disponible señala un incremento del riesgo de mortalidad asociado con la exposición prolongada a este metaloide ^4^^,^^6^. En América Latina, la presencia de arsénico en fuentes de agua ha sido ampliamente documentada, especialmente en regiones con actividad minera o con características geológicas volcánicas y sedimentarias. Revisiones regionales han descrito la existencia de áreas con concentraciones elevadas de arsénico en agua subterránea, afectando a poblaciones en países como Argentina, Chile, Bolivia, México y Perú. Estas condiciones han sido asociadas principalmente a procesos naturales de movilización geoquímica, así como a actividades antropogénicas, particularmente la minería ^7^^-^^9^.

En el Perú, la exposición a metales pesados y metaloides ha sido reconocida como un problema emergente de salud pública. Diversos estudios han documentado la presencia de arsénico en fuentes de agua destinadas al consumo humano, particularmente en zonas rurales y altoandinas. George *et al*. evidenciaron que una proporción importante de fuentes de agua en comunidades peruanas supera el valor guía provisional de 10 µg/L recomendado por la OMS para el arsénico en el agua de consumo, lo que representa un riesgo para la salud de las poblaciones expuestas ^10^. Asimismo, estudios en zonas mineras han mostrado la presencia de metales en biomarcadores humanos, lo que refleja exposición ambiental sostenida ^11^.

En el contexto andino, la exposición a arsénico adquiere particular relevancia debido a la combinación de factores ambientales, geológicos y socioeconómicos que condicionan el acceso a agua segura. En estas poblaciones, la dependencia de fuentes de agua no tratadas, junto con la proximidad a actividades mineras, puede incrementar el riesgo de exposición crónica. Estudios realizados en poblaciones andinas han reportado concentraciones elevadas de arsénico en fuentes de agua y en la orina, como biomarcador de exposición reciente ^12^.

Como antecedente local, en 2023 la Red de Servicios de Salud Chumbivilcas realizó una evaluación dirigida de exposición a metales en 80 pobladores de dos localidades previamente identificadas como de potencial riesgo: la zona de la cantera Llanque, en el distrito de Colquemarca, y el distrito de Quiñota. Mediante la determinación de arsénico en orina, 78 de las 80 personas evaluadas (97,5%) presentaron concentraciones ≥20 µg As/g de creatinina, tomando como referencia el valor establecido para personas no expuestas ocupacionalmente en la Guía Técnica para el Abordaje Integral a Personas Expuestas a Arsénico del Ministerio de Salud ^13^. La selección de las personas evaluadas no fue probabilística y estuvo focalizada en localidades previamente identificadas como de potencial riesgo, por lo que estos resultados constituyeron una señal sanitaria de exposición y no una estimación de prevalencia poblacional. En consecuencia, persistía la necesidad de estimar la magnitud de la exposición mediante un estudio probabilístico con cobertura provincial en la población de Chumbivilcas. En este contexto, el presente estudio tuvo como objetivo determinar la prevalencia y los factores asociados a la exposición elevada de arsénico en pobladores de la provincia de Chumbivilcas, Perú, durante el año 2024.

**MENSAJES CLAVE**

Motivación del estudio. En Chumbivilcas existe limitada evidencia poblacional basada en biomarcadores sobre la magnitud de la exposición a arsénico y sus factores asociados.

Principales hallazgos. Se encontró una elevada prevalencia de exposición a arsénico en la población adulta de Chumbivilcas. La exposición reportada en el trabajo y el uso de agua de manantial se asociaron con una mayor prevalencia.

Implicancias para salud pública. Los hallazgos respaldan la necesidad de fortalecer la vigilancia epidemiológica y ambiental en Chumbivilcas y profundizar la evaluación de potenciales fuentes de exposición, especialmente en el ámbito laboral y las fuentes de agua, mediante estudios que incorporen mediciones ambientales.

## MATERIALES Y MÉTODOS

### Diseño y población de estudio

Se realizó un estudio observacional transversal analítico. El estudio se desarrolló en la provincia de Chumbivilcas, ubicada en la región Cusco, en la zona sur del Perú, a una altitud promedio de 3,660 metros sobre el nivel del mar. Esta provincia presenta una geografía predominantemente rural y sus principales actividades económicas incluyen la agricultura, la ganadería, la minería y el comercio local. La población proyectada para el 2024 fue de 69,017 habitantes en sus 8 distritos ^14^. Se incluyeron participantes mayores de 18 años, de ambos sexos que residían en la zona por un periodo mínimo de seis meses previos a la recolección de datos; y se excluyeron aquellos que estuvieron fuera más de 10 días consecutivos en el último mes, con alguna discapacidad cognitiva, si habían consumido pescados o mariscos 3 días antes, y personas con enfermedad renal crónica o que desempeñen algún cargo político, público o representativo.

### Muestreo y tamaño de muestra

Se utilizó un muestreo probabilístico bietápico por conglomerados, estratificado por distritos de la provincia. En la primera etapa se seleccionaron los conglomerados poblacionales dentro de cada distrito, y en la segunda etapa se seleccionaron los hogares dentro de cada conglomerado. Posteriormente, se invitó a participar a los residentes adultos elegibles de los hogares seleccionados. El tamaño de muestra final estuvo conformado por 370 participantes en 25 conglomerados, con distritos con un conglomerado como Quiñota y otros con nueve conglomerados como Santo Tomás, quienes cumplieron los criterios de elegibilidad y aceptaron participar en el estudio.

### Variables del estudio

La variable dependiente fue la exposición a arsénico, determinada mediante la concentración de arsénico urinario ajustada por creatinina. Se consideró exposición cuando la concentración fue igual o mayor a 20 µg/g de creatinina, según criterios establecidos en la literatura internacional y adoptados por el Centro Nacional de Salud Ocupacional y Protección del Ambiente para la Salud (CENSOPAS) del Instituto Nacional de Salud (INS) ^15^.

Las variables independientes que, cumplen la función de factores sociodemográficos, epidemiológicos y ambientales se registraron mediante la Ficha de Investigación Epidemiológica en Salud Pública de Factores de Riesgo por Exposición e Intoxicación por Metales Pesados y Metaloides del Ministerio de Salud ^16^. La ficha registra la vía de exposición como respiratoria, oral, dérmica, desconocida u otra, y el tipo de exposición como ocupacional, accidental, ambiental u otro. Estas variables correspondieron a antecedentes epidemiológicos registrados mediante la ficha y no a mediciones directas de arsénico en matrices ambientales. Por otro lado, la cercanía de la vivienda a minería, relaves mineros y transporte de concentrados de metales correspondió al registro dicotómico (sí/no) establecido en la ficha, sin medición de una distancia específica. Asimismo, la presencia de polvo en el ambiente externo se registró de forma dicotómica (sí/no), sin medición instrumental de material particulado. Se definió el uso de alguna fuente de agua no potable como el reporte de consumo de agua de río, manantial o lluvia, independientemente del uso concomitante de fuentes potables.

### Procedimientos

El trabajo de campo se realizó durante siete días consecutivos, del 22 al 28 de julio de 2024, incluyendo días laborables y fin de semana. Las visitas domiciliarias se efectuaron entre las 6:30 y las 14:00 horas. En cada vivienda, el participante fue seleccionado al azar entre los adultos elegibles. Cuando la persona seleccionada no se encontraba en la vivienda o rechazaba participar, se seleccionó otro adulto elegible de la misma vivienda y, cuando ello no fue posible, un adulto de la vivienda contigua. La entrevista y la recolección de la muestra de orina se realizaron durante la misma visita domiciliaria.

Las muestras de orina fueron conservadas bajo refrigeración en el Hospital de Santo Tomás, Chumbivilcas, siguiendo las condiciones establecidas en el procedimiento del CENSOPAS-INS, que establece para la determinación de arsénico en orina puntual una temperatura de conservación entre 2 y 8 °C. Para su traslado desde Chumbivilcas hasta la ciudad de Cusco se utilizaron *coolers* con geles refrigerantes, manteniendo las muestras refrigeradas durante el transporte. Posteriormente, fueron transportadas por vía aérea desde Cusco hasta Lima para su recepción en CENSOPAS-INS. El envío se realizó en dos tandas, los días 25 y 29 de julio de 2024, durante el periodo de trabajo de campo, con la finalidad de mantener las muestras dentro de las condiciones y tiempos establecidos para su conservación.

Las muestras fueron procesadas en el Laboratorio Químico Toxicológico del CENSOPAS en Lima, Perú. La concentración de arsénico en orina se determinó mediante espectrometría de masas con plasma acoplado inductivamente (ICP-MS), utilizando el método MET-CENSOPAS-017 “Determinación de arsénico y cadmio en muestras de orina humana mediante espectrometría de masas con plasma acoplado inductivamente”. Los resultados fueron reportados en µg/L y normalizados por creatinina urinaria en µg As/g de creatinina.

### Análisis estadístico

Para el análisis se consideró el diseño muestral complejo del estudio, incorporando los ponderadores muestrales, la estratificación por distrito y la conglomeración. La prevalencia de exposición elevada a arsénico se estimó considerando el diseño muestral y se presentó con su intervalo de confianza al 95% (IC 95%). Para evaluar los factores asociados se estimaron razones de prevalencias (RP) crudas y ajustadas (RPa), con sus respectivos IC 95%, mediante modelos de regresión de Poisson con enlace logarítmico que incorporaron el diseño muestral. Las RPa para edad, sexo y procedencia se obtuvieron mediante un modelo mutuamente ajustado. Para las variables ocupacionales, epidemiológicas y ambientales se utilizaron modelos independientes ajustados por edad, sexo y procedencia, con el propósito de mantener modelos parsimoniosos en función de los grados de libertad disponibles del diseño muestral. Se consideró un valor de p<0,05 como estadísticamente significativo. El análisis estadístico se realizó utilizando R versión 4.5.2 (R Foundation for Statistical Computing, Vienna, Austria).

### Aspectos éticos

El estudio fue aprobado por el Comité de Ética en Investigación de la Universidad Nacional de San Antonio Abad del Cusco, mediante Informe N.º 007-2024.CBI-UNSAAC. Todos los participantes recibieron información sobre los objetivos del estudio y otorgaron su consentimiento informado previo a su participación. Asimismo, se garantizó la confidencialidad de la información recolectada y el uso de los datos exclusivamente con fines de investigación.

## RESULTADOS

Se incluyeron 370 participantes residentes en los ocho distritos de la provincia de Chumbivilcas. De ellos, 250 presentaron concentraciones de arsénico urinario ajustado por creatinina ≥20 µg As/g de creatinina y 120 presentaron valores por debajo de este punto de corte. Considerando el diseño muestral, la prevalencia ponderada de exposición elevada a arsénico fue de 68,2% (IC95%: 61,9-73,9). Las estimaciones puntuales de prevalencia ponderada por distrito variaron entre 56,9% en Santo Tomás y 86,7% en Capacmarca y Chamaca.

Las características de la población estudiada se presentan en la tabla 1. La edad media fue de 46,2 años (EE: 1,2); el 59,4% correspondió al sexo femenino y el 64,5% procedía del área rural. Respecto al nivel educativo, predominaron la educación secundaria (34,6%) y primaria (32,1%). Las ocupaciones más frecuentes fueron las actividades agropecuarias (36,1%) y las labores del hogar (34,2%).

**Tabla 1:** Características de la exposición a arsénico en adultos de la provincia de Chumbivilcas, Cusco 2024.

Características		n	(%)^a^
Edad, años^b^		370	46,2 (1,2)
Sexo			
	Femenino	217	(59,4)
	Masculino	153	(40,6)
Procedencia			
	Urbana	125	(32,6)
	Urbano-marginal	12	(2,9)
	Rural	233	(64,5)
Distrito^c^			
	Capacmarca	15	(86,7)
	Chamaca	30	(86,7)
	Colquemarca	45	(68,9)
	Livitaca	60	(76,7)
	Llusco	30	(60,0)
	Quiñota	15	(66,7)
	Santo Tomás	130	(56,9)
	Velille	45	(71,1)
Nivel de instrucción			
	Inicial	4	(1,1)
	Primaria	117	(32,1)
	Secundaria	131	(34,6)
	Superior	65	(17,2)
	Sin instrucción	53	(15,0)
Ocupación			
	Servidor público	29	(7,9)
	Independiente	48	(13,0)
	Estudiante	2	(0,4)
	Minería	32	(8,4)
	En su casa	125	(34,2)
	Agropecuario	134	(36,1)
Seguro de salud			
	SIS	353	(95,5)
	EsSalud	17	(4,5)
Fuente de agua^d^			
	Red	303	(83,0)
	Pileta	25	(6,3)
	Río	33	(8,6)
	Manantial	48	(12,1)
	Lluvia	7	(1,7)
	Alguna fuente no potable^e^	84	(21,5)
Concentración de arsénico urinario^f^			
	Media (EE)	327	39,39 (2,24)
	Mediana (RIC)	327	29,02 (20,44-44,75)
Exposición elevada a arsénico^g^			
	No	120	(31,8)
	Sí	250	(68,2)

a Las estimaciones incorporaron ponderación, estratificación y conglomeración del diseño muestral; ^b^ Media (error estándar); ^c^ Estimaciones puntuales de arsénico en los distritos. No se presentan intervalos de confianza debido al reducido número de conglomerados, lo que impidió su estimación con precisión confiable debido a su comparabilidad; ^d^ Las fuentes de agua no fueron mutuamente excluyentes; ^e^ Se consideró fuente no potable a agua reportada de agua de río, manantial o lluvia; ^f^ La concentración de arsénico fue cuantificable en 327 participantes y expresada en µg As/g de creatinina; la concentración de arsénico urinario fue cuantificada como media (error estándar) y como mediana (rango intercuartílico); ^g^ Se consideró exposición elevada una concentración urinaria ≥20 µg As/g de creatinina; los resultados no fueron estratificados como no expuestos.

Los resultados de los análisis ajustados no encontraron factores asociados con la exposición elevada a arsénico con las características sociodemográficos y ocupacionales asociados (tabla 2).

**Tabla 2:** Factores sociodemográficos y ocupacionales asociados con exposición elevada a arsénico en pobladores adultos de la Provincia de Chumbivilcas, Cusco 2024.

Características		RP cruda (IC95%)	“Modelo 1 RPa (IC95%)”	“Modelo 2 RPa (IC95%)”	“Modelo 3 RPa (IC95%)”
Edad, años		0,99 (0,98-1,02)	0,995 (0,98-1,00)		
Sexo					
	Femenino	Ref.	Ref.		
	Masculino	0,88 (0,77-1,02)	0,90 (0,78-1,05)		
Procedencia					
	Urbana	Ref.	Ref.		
	Urbano-marginal	1,13 (0,89-1,44)	1,12 (0,85-1,46)		
	Rural	1,23 (0,96-1,59)	1,26 (0,98-1,62)		
Ocupación					
	Servidor público	Ref.	-	Ref.	-
	Independiente	1,35 (0,90-2,04)	-	1,36 (0,92-2,00)	-
	Estudiante	1,02 (0,15-6,70)	-	0,90 (0,17-4,84)	-
	Minería	1,48 (0,92-2,38)	-	1,58 (0,96-2,59)	-
	Ama de casa	1,43 (0,90-2,27)	-	1,30 (0,84-2,01)	-
	Agropecuario	1,43 (0,89-2,31)	-	1,51 (0,95-2,39)	-
Actividad económica					
	Minería formal	Ref.	-	-	Ref.
	Minería informal	0,78 (0,66-0,94)	-	-	0,81 (0,66-0,98)
	Transporte de metales	0,87 (0,48-1,59)	-	-	0,92 (0,46-1,83)
	Soldaduría	0,60 (0,14-2,61)	-	-	0,76 (0,15-3,68)
	Agropecuario	0,74 (0,61-0,90)	-	-	0,78 (0,64-0,96)

Modelo 1 corresponde al ajuste mutuo entre edad, sexo y procedencia. Modelo 2 evalúa ocupación ajustando por edad, sexo y procedencia. Modelo 3 evalúa actividad económica ajustando por edad, sexo y procedencia. Todos los modelos incorporaron ponderación, estratificación y conglomeración del diseño muestral. Ref.: categoría de referencia.

En la tabla 3, se muestra que la exposición reportada en el trabajo se asoció con mayor prevalencia de exposición elevada a arsénico (RPa=1,29; IC95%: 1,07-1,56). En contraste, la vía de exposición respiratoria presentó una menor prevalencia (RPa=0,82; IC95%: 0,72-0,92). No se observaron asociaciones con las demás variables epidemiológicas evaluadas.

**Tabla 3:** Asociación entre indicadores epidemiológicos y exposición elevada a arsénico en pobladores adultos de la Provincia de Chumbivilcas, Cusco 2024.

Características		RP (IC95%)	RPa (IC95%)*
Exposición reportada en casa			
	No	Ref.	Ref.
	Sí	0,86 (0,73-1,01)	0,83 (0,69-0,99)
Exposición reportada en el trabajo			
	No	Ref.	Ref.
	Sí	1,21 (1,04-1,39)	1,29 (1,07-1,56)
Vía respiratoria reportada			
	No	Ref.	Ref.
	Sí	0,77 (0,66-0,90)	0,82 (0,72-0,92)
Vía oral reportada			
	No	Ref.	Ref.
	Sí	1,08 (0,93-1,25)	1,06 (0,84-1,34)
Vía desconocida			
	No	Ref.	Ref.
	Sí	0,92 (0,74-1,15)	0,98 (0,77-1,25)
Hábito de fumar			
	No	Ref.	Ref.
	Sí	1,17 (0,89-1,54)	1,14 (0,86-1,52)

* Ajustados por edad, sexo y procedencia. Todos los modelos incorporaron ponderación, estratificación y conglomeración del diseño muestral. La exposición en casa y en el trabajo corresponde a exposición reportada por los participantes. Las vías respiratoria, oral y desconocida corresponden a vías de exposición reportadas por los participantes. Ref.: categoría de referencia.

En la tabla 4, se muestra que el consumo de agua de manantial se asoció con mayor prevalencia de exposición elevada a arsénico (RPa=1,19; IC95%: 1,01-1,40). No se observaron asociaciones con el uso de alguna fuente de agua no potable ni con las demás variables ambientales evaluadas.

**Tabla 4:** Asociación entre factores ambientales y exposición elevada a Arsénico en pobladores adultos de la Provincia de Chumbivilcas, Cusco 2024.

Característica		RP (IC95%)	RPa (IC95%)^a^
Cercanía a actividad minera			
	No	Ref.	Ref.
	Sí	1,07 (0,90-1,26)	1,04 (0,90-1,21)
Cercanía a relave minero			
	No	Ref.	Ref.
	Sí	1,06 (0,80-1,41)	1,01 (0,74-1,37)
Cercanía a transporte de metales/minerales			
	No	Ref.	Ref.
	Sí	1,22 (0,75-1,98)	1,20 (0,72-2,02)
Cercanía al corredor			
	No	Ref.	Ref.
	Sí	0,86 (0,63-1,19)	0,82 (0,60-1,13)
Presencia de polvo en el exterior			
	No	Ref.	Ref.
	Sí	0,93 (0,54-1,59)	0,97 (0,58-1,63)
Uso de alguna fuente de agua no potable			
	No	Ref.	Ref.
	Sí	1,04 (0,81-1,34)	0,99 (0,78-1,24)
Uso de agua de manantial			
	No	Ref.	Ref.
	Sí	1,25 (1,05-1,49)	1,19 (1,01-1,40)

a Ajustados por edad, sexo y procedencia. Todos los modelos incorporaron ponderación, estratificación y conglomeración del diseño muestral. La fuente de agua no potable se definió como el uso reportado de al menos una de las siguientes fuentes: río, manantial o agua de lluvia; las fuentes de agua no fueron mutuamente excluyentes. El uso de agua de manantial se evaluó adicionalmente como fuente específica de agua. Ref.: categoría de referencia.

## DISCUSIÓN

En este estudio poblacional realizado en la provincia de Chumbivilcas, se encontró una elevada prevalencia ponderada de exposición a arsénico (68,2%; IC95%: 61,9-73,9). La exposición reportada en el trabajo y el uso de agua de manantial se asociaron con una mayor prevalencia de exposición elevada a arsénico. En contraste, la vía respiratoria registrada presentó una asociación inversa. No se encontró asociación con el uso de alguna fuente de agua no potable ni con la ocupación.

La magnitud de exposición observada en Chumbivilcas es consistente con la heterogeneidad descrita en América Latina, donde la contaminación por arsénico afecta tanto aguas subterráneas como superficiales y presenta importantes variaciones geográficas, particularmente en regiones andinas con condiciones geológicas y actividades mineras que pueden favorecer su movilización ^1^^,^^2^.

En Perú, Ale-Mauricio *et al*. reportaron en Cairani y Camilaca, Tacna, medianas de arsénico urinario de 601,6 y 30,2 µg/g de creatinina, respectivamente, con 100% y 80,3% de los participantes por encima de 20 µg/g de creatinina; en estos distritos, el abastecimiento de agua fue descrito como de origen subterráneo, con aportes del río Callazas y del manantial Japo ^17^. Asimismo, Fano-Sizgorich *et al*. encontraron en gestantes de Tacna una media de arsénico urinario total de 43,97 µg/L y señalaron que, en una evaluación previa de esta población, 70,2% estuvo expuesta a concentraciones de arsénico en agua de consumo ≥25 µg/L ^18^.

En Bolivia, De Loma *et al*. reportaron una mediana de arsénico urinario de 65 µg/L en mujeres de comunidades próximas al lago Poopó, donde se documentaron concentraciones elevadas de arsénico en fuentes de agua de consumo ^12^. Estas diferencias entre poblaciones podrían relacionarse con la heterogeneidad geológica de la región andina, las fuentes y magnitud de exposición y las características de las poblaciones estudiadas ^1^^,^^2^^,^^12^. En Chumbivilcas, aunque el uso de agua de manantial se asoció con mayor prevalencia de exposición elevada a arsénico, el uso de alguna fuente de agua no potable no mostró asociación; por ello, nuestros resultados no permiten atribuir la elevada prevalencia observada al agua de consumo en general.

La magnitud de exposición observada fue menor que la reportada en poblaciones específicamente seleccionadas por su exposición ambiental, lo que debe interpretarse considerando el enfoque poblacional de nuestro estudio. La exposición elevada a arsénico estuvo presente tanto en participantes que reportaron residir cerca de actividad minera como en quienes no lo hicieron, sin evidenciarse asociación con esta condición.

La exposición reportada en el trabajo se asoció con una mayor prevalencia de exposición elevada a arsénico (RPa=1,29; IC95%: 1,07-1,56), lo que es compatible con la evidencia que reconoce los ambientes ocupacionales y las actividades vinculadas a minería y metales como posibles escenarios de exposición ^1^^,^^2^^,^^15^^,^^19^. En contraste, la exposición reportada en casa mostró una asociación inversa (RPa=0,83; IC95%: 0,69-0,99). Este resultado debe interpretarse con cautela, pues ambas variables corresponden a antecedentes registrados mediante la ficha epidemiológica y no a mediciones ambientales de arsénico; por ello, no permiten identificar el lugar ni la fuente específica de exposición.

El uso de agua de manantial se asoció con una mayor prevalencia de exposición elevada a arsénico (RPa=1,19; IC95%: 1,01-1,40). Este hallazgo es consistente con la relevancia de las fuentes hídricas como vía de exposición descrita en el Perú y otros contextos andinos. George *et al.* documentaron concentraciones superiores a 10 µg/L de arsénico en el 86% de las muestras de agua subterránea evaluadas en diferentes localidades del Perú ^10^. Asimismo, García-Rico *et al.* documentaron arsénico en agua de consumo y suelos de comunidades de Sonora, México, y encontraron una correlación positiva entre los niveles de arsénico urinario y el índice de riesgo ambiental ^20^; En el Altiplano chileno se han descrito concentraciones elevadas de arsénico en aguas superficiales, relacionadas con las características geoquímicas de la región ^21^. La presencia de arsénico en fuentes hídricas de América Latina puede obedecer a diversos procesos geogénicos y antropogénicos, con particular relevancia de las condiciones geológicas andinas ^1^. Sin embargo, en nuestro estudio el uso de alguna fuente de agua no potable no mostró asociación con la exposición elevada a arsénico, por lo que el hallazgo específico para agua de manantial no permite establecer que el agua sea la fuente causal de exposición, dado que no se midieron concentraciones de arsénico en las fuentes de agua utilizadas por los participantes.

La vía respiratoria registrada en la ficha epidemiológica se asoció con una menor prevalencia de exposición elevada a arsénico (RPa=0,82; IC95%: 0,72-0,92). Este hallazgo debe interpretarse con cautela y no implica un efecto protector de la exposición respiratoria. La variable correspondió al antecedente de vía de exposición consignado mediante la ficha epidemiológica y no a una medición de arsénico en el aire ni a una evaluación cuantitativa de la exposición por inhalación. Aunque la inhalación puede constituir una vía relevante de exposición al arsénico en determinados contextos ocupacionales y ambientales ^1^^,^^2^^,^^22^, nuestro estudio no permite establecer un mecanismo que explique la asociación inversa observada. Por ello, este resultado podría reflejar diferencias en los perfiles o fuentes de exposición de los participantes y requiere ser evaluado en estudios que incorporen mediciones ambientales específicas.

Las diferencias observadas entre nuestros resultados y los reportados en otros estudios pueden explicarse por variaciones en las condiciones hidrogeológicas, los tipos de biomarcadores utilizados, los puntos de corte empleados y las características de las poblaciones evaluadas. Además, los estudios realizados en zonas específicas con alta contaminación tienden a reportar niveles más elevados de exposición en comparación con estudios poblacionales.

Desde el punto de vista de la salud pública, nuestros hallazgos son relevantes debido a las múltiples consecuencias sanitarias asociadas con la exposición crónica a arsénico. La evidencia epidemiológica ha demostrado que la exposición prolongada se asocia con mayor riesgo de cáncer, enfermedades cardiovasculares y alteraciones neurológicas ^4^^-^^6^. En este contexto, una prevalencia elevada como la observada en nuestra población sugiere un riesgo potencial de carga de enfermedad crónica acumulativa.

Este estudio presenta algunas limitaciones. En primer lugar, no se realizaron mediciones de arsénico en agua, aire, suelo, alimentos u otras matrices ambientales, por lo que las asociaciones observadas no permiten identificar la fuente específica de exposición. Las variables epidemiológicas y ambientales se obtuvieron mediante la ficha de investigación y no representaron mediciones cuantitativas de la intensidad o dosis de exposición, por lo que son susceptibles a errores de clasificación y sesgo de información. Finalmente, el número limitado de conglomerados redujo los grados de libertad disponibles para el análisis y restringió la complejidad de los modelos multivariables; en consecuencia, se utilizaron modelos parsimoniosos independientes ajustados por edad, sexo y procedencia, sin que pueda descartarse confusión residual. Esta limitación también afectó la precisión de las estimaciones por distrito.

Aunque el trabajo de campo incluyó días laborables y fines de semana y las visitas se iniciaron desde las 6:30 horas, lo que amplió la oportunidad de incluir personas laboralmente activas antes del inicio de sus actividades habituales, el reemplazo de participantes seleccionados que se encontraban ausentes o rechazaron participar por otros adultos de la misma vivienda o, cuando ello no fue posible, de una vivienda contigua pudo modificar las probabilidades individuales de selección y generar sesgo de selección. No es posible establecer la dirección ni la magnitud de este potencial sesgo sobre las estimaciones obtenidas.

Entre las fortalezas del estudio destaca su enfoque poblacional, con inclusión de participantes de los ocho distritos de la provincia de Chumbivilcas mediante un muestreo probabilístico por conglomerados estratificado por distrito. El análisis incorporó la ponderación, estratificación y conglomeración del diseño muestral, permitiendo obtener estimaciones poblacionales y considerar la estructura de la muestra en la estimación de su precisión. Asimismo, la exposición elevada se determinó mediante la medición de arsénico urinario, proporcionando una medida objetiva del biomarcador de exposición. Finalmente, la evaluación conjunta de características sociodemográficas y epidemiológicas y ambientales permitió explorar diferentes factores en la población general y generar evidencia para orientar posteriores investigaciones ambientales y epidemiológicas.

En conclusión, en la población estudiada de Chumbivilcas se encontró una elevada prevalencia ponderada de exposición a arsénico. La procedencia mostró una asociación global con la exposición elevada, mientras que la exposición reportada en el trabajo y el uso de agua de manantial se asociaron con una mayor prevalencia; en contraste, la exposición reportada en el hogar y la vía respiratoria mostraron asociaciones inversas que deben interpretarse con cautela. Estos hallazgos no permiten establecer fuentes ni vías causales de exposición y evidencian la necesidad de fortalecer la vigilancia epidemiológica y ambiental, así como desarrollar estudios que integren biomonitoreo y mediciones de arsénico en agua y otras matrices ambientales para identificar las fuentes de exposición y orientar medidas de prevención en Chumbivilcas.
